# Enhancing Crop Domestication Through Genomic Selection, a Case Study of Intermediate Wheatgrass

**DOI:** 10.3389/fpls.2020.00319

**Published:** 2020-03-24

**Authors:** Jared Crain, Prabin Bajgain, James Anderson, Xiaofei Zhang, Lee DeHaan, Jesse Poland

**Affiliations:** ^1^Department of Plant Pathology, Kansas State University, Manhattan, KS, United States; ^2^Department of Agronomy and Plant Genetics, University of Minnesota, St. Paul, MN, United States; ^3^The Alliance of Bioversity International and International Center for Tropical Agriculture, Cali, Colombia; ^4^The Land Institute, Salina, KS, United States

**Keywords:** intermediate wheatgrass, genomic selection, multi-environment, domestication, perennial crops, shared data resources

## Abstract

Perennial grains could simultaneously provide food for humans and a host of ecosystem services, including reduced erosion, minimized nitrate leaching, and increased carbon capture. Yet most of the world’s food and feed is supplied by annual grains. Efforts to domesticate intermediate wheatgrass (*Thinopyrumn intermedium*, IWG) as a perennial grain crop have been ongoing since the 1980’s. Currently, there are several breeding programs within North America and Europe working toward developing IWG into a viable crop. As new breeding efforts are established to provide a widely adapted crop, questions of how genomic and phenotypic data can be used among sites and breeding programs have emerged. Utilizing five cycles of breeding data that span 8 years and two breeding programs, University of Minnesota, St. Paul, MN, and The Land Institute, Salina, KS, we developed genomic selection (GS) models to predict IWG traits. Seven traits were evaluated with free-threshing seed, seed mass, and non-shattering being considered domestication traits while agronomic traits included spike yield, spikelets per inflorescence, plant height, and spike length. We used 6,199 genets – unique, heterozygous, individual plants – that had been profiled with genotyping-by-sequencing, resulting in 23,495 SNP markers to develop GS models. Within cycles, the predictive ability of GS was high, ranging from 0.11 to 0.97. Across-cycle predictions were generally much lower, ranging from −0.22 to 0.76. The prediction ability for domestication traits was higher than agronomic traits, with non-shattering and free threshing prediction abilities ranging from 0.27 to 0.75 whereas spike yield had prediction abilities ranging from −0.22 to 0.26. These results suggest that progress to reduce shattering and increase the percent free-threshing grain can be made irrespective of the location and breeding program. While site-specific programs may be required for agronomic traits, synergies can be achieved in rapidly improving key domestication traits for IWG. As other species are targeted for domestication, these results will aid in rapidly domesticating new crops.

## Introduction

Currently, 80% of the world’s calories are provided by annual crops (Pimentel et al., 2012), with only three crops, maize (*Zea mays*), wheat (*Triticum aestivum*), and rice (*Oryza sativa*), providing nearly 60% of human calorie consumption (fao.org). Additionally, 70% of arable land is planted to annual crops (Cox et al., 2010; Pimentel et al., 2012) that are resource-intensive and can result in environmental degradation (Power, 2010; Crews et al., 2018). Perennial grain crops could provide abundant ecosystem services while simultaneously providing food, feed, and fuel for the global population. To date, there are no widely planted perennial grain crops, but recent research has resulted in large scale evaluations of perennial rice (Huang et al., 2018) and perennial versions of several other crops including wheat, sorghum (*Sorghum bicolor*), sunflower (*Helianthus*), and pulses are in development (Batello et al., 2013).

One species showing promise for domestication and wide-scale production is intermediate wheatgrass (*Thinopyrum intermedium*, IWG). Intermediate wheatgrass is native to Eastern Europe and the Mediterranean region (Tsvelev, 1983) and was introduced into the United States for erosion control and forage purposes in 1932 (Vogel and Jensen, 2001). This species was selected for domestication as a grain crop in the 1980’s from an evaluation of nearly 100 perennial grasses based on its seed size, vigorous growth habit, and potential for mechanical harvest, among other desirable characteristics, at the Rodale Institute, Kutztown, PA (Wagoner, 1990). In the early 2000’s after two cycles of selection at the USDA’s Big Flats Plant Materials Center, Corning, NY, breeding efforts shifted to The Land Institute (TLI), Salina, KS (Zhang et al., 2016). Since 2003, nine cycles of selection have been completed at TLI. Interest in IWG has led to the development of several other breeding programs, including the University of Minnesota (UMN) and University of Manitoba in 2011 using material from the third cycle of selection from TLI (TLI-C3) (Zhang et al., 2016). Products made from IWG grain are being sold under the trade name Kernza in limited markets (DeHaan and Ismail, 2017).

Along with intensive breeding effort, IWG has also been evaluated for a host of ecosystem services. Research has shown that IWG can reduce soil nitrate leaching by 86% or more compared to annual wheat crop systems (Culman et al., 2013). Jungers et al. (2019) found that nitrate leaching under perennial grasses, including IWG, were one to two orders of magnitude less than annual maize. IWG has also been reported to have 15 times more root growth and nearly two times the above-ground biomass of annual wheat (Sprunger et al., 2018), which should translate into greater below-ground carbon storage rates. Research also indicates that perennial landscapes have significantly increased and diverse microbial communities, allowing for greater food web complexity and increased nutrient cycling capacity (Culman et al., 2010; Pugliese et al., 2019). While IWG has the potential to provide both food and ecosystem services, factors such as grain yield and ability to mechanically harvest must be improved to an economically viable level for farmers to adopt this new crop.

Breeding new crops from wild species requires domestication, which often utilizes rare allelic mutations to facilitate the development of crops. One common domestication trait has been the prevention of shattering, which enables mechanical harvest. Numerous domestication events have been recorded in barley, rice, and sorghum (Østerberg et al., 2017), with reduction of shattering a hallmark of domestication as the plant becomes more dependent on humans for seed dispersal (Purugganan and Fuller, 2009). Other key traits that have evolved through domestication include larger seed size, free threshing seeds, and an increase in percent seed set (Harlan et al., 1973). Within IWG breeding, key domestication traits being targeted are greater percent of free threshing seeds, reduction in seed shattering, and increased seed mass.

Early work in domestication architecture through quantitative trait loci (QTL) often suggested single or a few genes with large effects (Koinange et al., 1996; Olsen and Wendel, 2013), which would allow for more efficient selection than selection on numerous loci with small effects (Falconer and Mackay, 1996). As molecular tools and studies have improved, there has been increasing evidence that many domestication traits are controlled by numerous loci with small effects. While the exact number of domestication genes is unknown (Meyer and Purugganan, 2013), in maize one study has identified nearly 500 genomic regions that had been under selection for domestication features (Hufford et al., 2012).

While original IWG breeding work utilized recurrent phenotypic selection, modern genetic tools have provided breeders with new options. One of the most promising genetic tools for breeding is genomic selection (GS). Proposed by Meuwissen et al. (2001), GS functions by having dense marker coverage of the entire genome so that each QTL is in linkage disequilibrium with a marker (Goddard and Hayes, 2007). Using a population that has been both phenotyped and genotyped, a model can be developed to predict the phenotype of individuals that have only been genotyped. GS has been shown to increase the rate of genetic gain in animal and plant breeding (Bernardo and Yu, 2007; García-Ruiz et al., 2016; Crossa et al., 2017). Given the predicted benefits of GS, Zhang et al. (2016) evaluated the potential of GS in IWG for the UMN breeding program, and currently, TLI is primarily using GS within their IWG breeding program.

While multiple locations are breeding IWG, there has been limited integration of information between breeding programs. The opportunity to utilize molecular tools like GS across wide environments could open new potentials for faster genetic improvement, specifically by increasing the training population size (VanRaden et al., 2009), integrating more genotypes (Knapp and Bridges, 1990), and taking advantage of correlated environments (Spindel and McCouch, 2016). Genomic selection has been used to improve a variety of polygenic agronomic traits including yield, quality and disease resistance (Rutkoski et al., 2014; Battenfield et al., 2016; Guzman et al., 2016). For crop wild relatives undergoing domestication, there has been less work on the ability of GS to improve key domestication traits such as shattering and free threshing. Work by Zhang et al. (2016) suggested that GS could be used to improve free threshing in IWG.

Applying GS to IWG across multiple environments could be a very cost-effective and efficient method to increase IWG breeding gains, but even within annual crops there is limited information about multi-site or multi-environment GS compared to single site GS studies. Lopez-Cruz et al. (2015) found that using marker-by-environment interactions resulted in a greater prediction accuracy than using within-environment models. A reaction norm model was used to generate prediction accuracies up to 0.4 in wheat in different environments throughout Kansas (Jarquín et al., 2017). In barley (*Hordeum vulgare*), a multi-environment GS model was shown to increase prediction accuracy 11% over single-environment analysis (Oakey et al., 2016). Resende et al. (2012) found that prediction accuracies in loblolly pine (*Pinus taeda*) were relatively consistent across environments as long as the environments were within the same breeding zone. However, in both of these examples many of the lines had true replication, whereas the IWG programs usually have single genotypes due to the challenges of cloning large numbers of individuals. As IWG breeding expands, the ability to combine data across multiple locations and breeding programs with differing, unreplicated, germplasm could be beneficial to increasing the rate of genetic gain.

Given the need for new crops and the challenges associated with developing perennial crops, this study focused on (1) How data from diverse sites and breeding programs could be combined to improve prediction abilities of models for enhanced selection decisions, (2) The ability of GS to accurately predict traits across a range of environments and traits, with emphasis on differences between domestication and agronomic traits, and (3) How insights gained from IWG breeding could be applied to other potential new crops undergoing domestication.

## Materials and Methods

### Plant Material and Field Establishment

Using terminology consistent with Zhang et al. (2016), we refer to a genet as a unique individual plant with its own genetic makeup. The genets used for this study consisted of the TLI Cycles 6, 7, and 8 (TLI-C6, TLI-C7, TLI-C8) and UMN Cycles 1 and 2 (UMN-C1, UMN-C2) breeding programs. The IWG TLI-C6 consisted of 3,658 genets from 674 full-sib families grown in one site location at Salina, KS (38.7684° N, 97.5664° W) between 2015 and 2017. Genets were established in the fall of 2015 with 91 cm between rows and 61 cm between columns, and phenotypic evaluations were conducted in 2016 and 2017. DeHaan et al. (2018) provide additional details about the TLI-C6 population. TLI-C7 was formed from random intermating between selected TLI-C6 genets. Genomic selection was used in the TLI-C7 generation, and a training population consisting of 1,179 genets from approximately 4,000 genotyped genets, was planted in the fall of 2017. TLI-C8 genets were progeny from selected TLI-C7 individuals and consisted of 988 selected, training population genets from approximately 3,500 genotyped genets, with field planting occurring in the fall of 2018. Both TLI-C7 and C8 were divided into two groups with approximately half of each cycle being planted in an irrigated field, and the other half in a non-irrigated field, providing two contrasting environments for evaluation.

UMN-C1 consisted of 2,560 genets from 66 half-sib families from TLI-C3 material. Genets were established in the field, St. Paul, MN (44.9906° N, 93.1799° W) in the fall of 2011 with field observations in 2012 and 2013. Additional information about the UMN-C1 population can be found in Zhang et al. (2016). The UMN-C2 training population consisted of 372 genets that were established in the fall of 2014 with observations in 2015 and 2016. UMN-C2 was obtained from open-pollination of 48 genets selected from the UMN-C1 population with the best agronomic performance. UMN-C2 consisted of 1,656 genets, but phenotypic observations were only recorded for 372 genets, the training population for GS within the UMN breeding program. In both cycles, genets were planted in a single replication at a distance of 1 m rows and columns, 67 kg ha^–1^ of N was applied in April of each year. Weed control in the plant nurseries was primarily done manually with a one-time application of herbicide Dual II Magnum (S-Metolachlor 82.4%, Syngenta) in April at a rate of 1.2 L ha^–1^. Experimental genets were surrounded on all sides with IWG plants. While each program is selecting genets for its respective growing region, all original UMN material, i.e., UMN-C1, came from TLI-C3, providing a common genetic link between the programs. All genets were evaluated as single plants with no replication.

### Field Evaluations

Field evaluations were completed for several key domestication and agronomic traits including: plant height, spikelets per inflorescence, spike length, spike yield, shattering, seed mass, and free-threshing. Plant height was measured after plants reached physiological maturity and was measured from the ground to the tip of the tallest spike. Shattering was measured on a five point scale, with 0 representing no shattering and 4 representing over 50% shattering by visual observation (DeHaan et al., 2018). Spike length was measured from the peduncle to the tip of the spike, and spikelets per inflorescence represented the average number of spikelets per head. Not all traits were measured for each year and genet, resulting in an unbalanced data set. Of key domestication traits, shattering was the only trait not observed in UMN-C1 and seed mass was not available for UMN-C2; all other traits were recorded in all cycles. In addition, minor differences in data collection between programs were noted. For UMN-C1 free threshing was measured on a four-point categorical scale, while for other years free threshing was estimated on a 0–100 percentage scale. The four-point scale was translated to match the percentage scale. For TLI cycles, spike yield was the mass of clean seed from one head, whereas in UMN cycles spike yield was estimated by weighing the entire seed head. Trait data was measured for 2 years with each year being considered a separate trait, with the exception of TLI-C8 with first year phenotypic data being recorded in 2019.

### Genotyping and Bioinformatic Methods

All genets were profiled using genotyping-by-sequencing following protocols of Poland et al. (2012) using a two enzyme restriction digest with *Pst*I and *Msp*I. Libraries were prepared by multi-plexing 192 samples per GBS library, and all GBS libraries were sequenced on Illumina HiSeq 2500. Single nucleotide polymorphisms (SNPs) were called using the GBS pipeline in Trait Analysis by aSSociation, Evolution, and Linkage (TASSEL) software version 5.2 (Glaubitz et al., 2014) in association with the IWG reference genome (access provided by the *Thinopyrum intermedium* Genome Sequencing Consortium.^1^

Initial SNP discovery resulted in identifying 126,138 SNPs. To identify a final data set, filtering was completed using the following criteria, (1) minor allele frequency greater than 0.01, (2) each SNP was called in 30% or more of the individuals, (3) GBS tags uniquely aligned (one location) to the reference genome to prevent aligning to orthologous sequences, (4) only biallelic SNPs were retained, (5) a minimum read depth of four tags per individual were required to call a homozygote. Using a custom Perl script, homozygotes that had less than four reads per site were set to missing. Heterozygotes were called with a minimum of two contrasting tags. Additionally, any genet that had more than 95% missing SNPs calls was discarded from the analysis, resulting in a final data set of 23,495 SNP loci and 6,199 genets. Any missing genotype calls in the final data set were imputed using Beagle version 4.1 using the default settings (Browning and Browning, 2016).

The STRUCTURE program (Pritchard et al., 2000) was used to evaluate population structure among the 6,199 genets. A subset of 8,011 markers that had minor allele frequency greater than 0.05 and were present in more than 50% of the individuals were used to evaluate population structure. A total of 10 subgroups (*K* = 1–10) were evaluated using the admixture model with 100,000 reps and the first 25,000 as burn-in. Ten replicates of each value of K were assessed, with Structure Harvester (Earl and vonHoldt, 2012) used to determine the optimal number of K. CLUMPP (version 1.1.2) (Jakobsson and Rosenberg, 2007) was used to evaluate *K* = 1 and *K* = 2 through graphically assigning individuals to a cluster. In addition to STRUCTURE, principal component analysis (PCA) was performed on the imputed marker matrix in R (R Core Team, 2017). The PCA results were used to subset genets into two similarity groups based on breeding programs.

### Statistical Analysis

A mixed linear model using ASREML version 4.1 (Gilmour et al., 2015) was fit to the data to develop best linear unbiased predictors (BLUPs) for each genet in each cycle. The model consisted of a two-step model, where each cycle was analyzed separately (Piepho et al., 2012), and BLUPs were then combined for GS. The model accounted for the genetic relationships between genets using the realized additive genomic relationship matrix and spatial location by fitting a separate row and column autoregressive order 1 (AR1 × AR1) residual structure for each site. The general form of the mixed model is (Isik et al., 2017):

(1) y=Xb+Zu+e

where ***y*** is a vector of observed phenotypes, **X** and **Z** are design matrices for fixed and random effects, respectively, ***b*** and ***u*** are vectors of coefficients for fixed and random effects, and ***e*** is a vector of random residuals. The vector ***y*** is assumed to be distributed normally with mean ***Xb*** and variance **V**, ***y*∼** *N(****Xb, V***). The total variance, **V**, is defined as $V=(\begin{array}{c} u\\ e \end{array})=\left(\begin{array}{cc} G & 0\\ 0 & R \end{array}\right)$. The **G** structure accounts for the variation between genets using the realized additive genomic relationship matrix and is defined as **G** = $\sigma_{A}^{2}$**K** where $\sigma_{A}^{2}$ is the additive genetic variance and **K** is the realized additive genomic relationship matrix. **K** is computed as θ**MM**’ where **M** is a matrix with n individuals and m columns of markers and θ is a proportionality constant (Endelman and Jannink, 2012). The genomic relationship matrix was computed using the function *A.mat* in *rrblup* (Endelman, 2011) R package using the methods of Endelman and Jannink (2012). The **R** structure accounts for residual variation using the row-column design for each cycle. The **R** for each site was defined as **R =** $\sigma_{e}^{2}\,\Sigma_{c}\,\left(\rho_{c}\right)⊗\sigma_{e}^{2}\,\Sigma_{r}\,\left(\rho_{r}\right)$, fitting an AR1 row and AR1 column effect with an independent error variance for each site. A total of seven sites were fit, as TLI-C7 and TLI-C8 each had two separate locations, whereas all other cycles were grown in one location. Σ is an identity matrix with dimensions equal to the number of rows or columns (Σ_*r*_, Σ_*c*_) respectively and ρ is the correlation parameter between rows and columns, respectively. A minimum of 350 observations were recorded from each cycle for use in GS models after adjusting phenotypic data for genetic relationship and spatial location in the field.

### Genomic Selection

Using the five cycles of data, GS models using the genomic best linear unbiased predictor (GBLUP) were developed to assess prediction ability. Within each cycle, a fivefold cross-validation method was repeated 100 times. For each iteration of the cross-validation, we randomly sampled all of the genets that were in a given cycle, splitting the genets into a training population (80% of genets) and a prediction population (20% of genets). The GS model was fit with the training population using *rrBLUP kin.blup* function (Endelman, 2011), with predictions then being made on the prediction population. The GS model has the form (Endelman, 2011):

(2) y=Wg+e

where ***y*** is a vector of observations (phenotypic BLUPs, section Statistical Analysis), ***W*** is a design matrix relating genets to observations, ***g*** is a vector of genotypic values, and ***e*** is a vector of random residuals. The vector of genotypic values, ***g***, is distributed as g ∼N(0, **K**$\sigma_{g}^{2}$), where **K** is the realized additive relationship matrix and $\sigma_{g}^{2}$ is the additive genotypic variance.

For each iteration, a random sampling without replacement was used to divide the training and prediction populations. Additionally, the random sampling did not prevent full or half-siblings from being both in the training and prediction populations, potentially upwardly biasing predictions. Predictive ability was assessed using Pearson correlation between the predicted value (genomic BLUP, GBLUP) and the BLUP for the respective phenotype. From the GS model, variance components were extracted to calculate genomic heritability using the genetic variance and residual error variance using the formula (Endelman and Jannink, 2012):

(3) $$h^{2}=\frac{\sigma_{a}^{2}}{\sigma_{a}^{2}+\sigma_{e}^{2}}$$

where *h*^2^ is narrow-sense heritability, $\sigma_{a}^{2}$ is genetic variance, and $\sigma_{a}^{2}+\sigma_{e}^{2}$ is the sum of genetic and residual variance representing total phenotypic variance.

To evaluate multi-environment predictions, each cycle was used as the training population to predict all other cycles. In this method, each cycle was fit as the training population, and then all other cycle genets were predicted. Using BLUPs for observed traits, accuracy was considered the correlation between the phenotypes and the GBLUPs, with the 95% confidence intervals for the correlation computed using the *psychometric* R package (Fletcher, 2010). Along with predicting all other sites from each site, a model was evaluated with a leave-one-out strategy, where the training population consisted of four cycles, and the final cycle was predicted from the combined training population.

**FIGURE 1:**
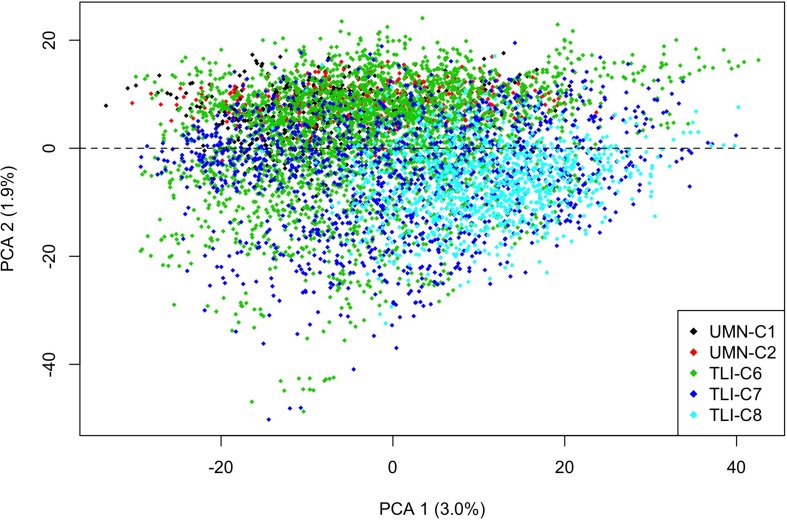
Scatterplot of the first two principal component axis for intermediate wheatgrass genets, made from principal component analysis on the marker matrix, *n* = 6,199 genets, markers = 23,495. Each point is an individual genet that is color coded by cycle, with the 2nd principal component providing separation between the UMN and TLI breeding programs at the dashed line. Total variance explained by each principal component is listed on the axis.

Two other models were developed with the goal of identifying the best ways to use the data sets to increase genetic gain. A subset of data was made using the results of the PCA analysis to create two similar groups, UMN-PCA and TLI-PCA. These models used the 2nd principal component to divide UMN and TLI material (Figure 1), with training data only consisting of genets within a respective group. In addition, to developing training sets by genetic similarity, each individual breeding program was used as a prediction set to predict all other cycles. The multi-environment models, where one cycle was predicted from all others, were ran again using these two data subsets to evaluate the effect of using more related training data sets in the prediction model. A minimum of 100 genets were required to be in the training set to make predictions for each model.

## Results

### Phenotypic Evaluations

We analyzed 8 years of breeding program field trials representing two independent breeding programs and five cycles of selection. Across all sites, several traits were measured, including the key domestication traits of free threshing and shattering and agronomic traits like spike yield and seed mass (Table 1). For all of these traits, a large range in observations were observed in all cycles. For example, individuals in most cycles ranged from no shattering to maximum shattering. For agronomic traits, a two or threefold range was present for spike length and spikelets per infloresence (Table 1).

**TABLE 1:** Range of phenotypic observations collected for five cycles of intermediate wheatgrass breeding trials from the University of Minnesota and The Land Institute.

		Free threshing %		Plant height (cm)		Seed Mass (mg)		Shattering 0–4		Spikelets per Inflorescence		Spike length (cm)		Spike yield (g)	
Cycle	Year	n	Range	n	Range	n	Range	n	Range	n	Range	n	Range	n	Range
UMN-C1	2012	494	3–75^†^	494	61–170	494	3.7–13.4			494	9–32	494	17–38	494	0.4–2.2^‡^
	2013	477	3–75^†^	491	76–185	474	3.7–13.9			484	10–32	485	17–36	484	0.1–2.0^‡^
UMN-C2	2015	372	0–95	372	70–182			372	0–4			372	13–41	372	0.4–2.0^‡^
	2016	356	0–80	368	104–190			360	0–4			367	19–37	366	0.4–1.7^‡^
TLI-C6	2016	2496	0–100	2482	30–180	2494	0.8–13.7	2507	0–4	2507	9–43	2506	13–60	2508	0.0–0.8
	2017	1691	0–100	1278	80–170	1690	2.5–13.3	1719	0–5	1714	12–39	1707	12–53	1723	0.0–0.7
TLI-C7	2018	851	0–100	1179	35–160	848	4.0–14.6	1140	0–4	1139	8–32	1141	0–52	1140	0.0–0.9
	2019	1164	0–100	1147	80–190	1162	2.9–17.2	1167	0–4	1168	11–32			1168	0.0–1.2
TLI-C8	2019	872	4–100	961	40–140	867	4.8–16.3	873	0–4	873	11–31			870	0.0–1.1

*Range of phenotypic observations and the number of individuals (n) for each phenotype are displayed. ^†^Trait was measured on a five-point categorical scale and converted to percentages. ^‡^Spike yield for UMN sites measured as entire inflorescence, not just clean seed.*

### Population Structure

We implemented a Bayesian cluster method to estimate population structure. While all genets were derived from TLI breeding material, this study evaluated five cycles of selection at different locations, times, and generations from the base population, allowing for potential population structure. Results from this analysis suggested that there was no population grouping of genets. Further analysis using PCA confirmed minimal population structure as the first principal component contained 3% of the variation and the first 10 components only accounted for 13% of the total variation. There was minor clustering among cycles (Figure 1), with the second principal component partially separating the UMN material and later TLI-C6 and C7 breeding programs.

### Genomic Selection Models

#### Within-Cycle Predictive Ability

To evaluate the potential of GS to increase the rate of genetic gain in IWG breeding, we fit several GS models to the phenotypic BLUPs. To determine predictive ability of GS, we fit a random fivefold cross-validation model to each cycle and trait individually. Using 100 iterations, within-cycle prediction ability, correlation between predicted value and the phenotypic BLUP, ranged from 0.11 to 0.97 (Table 2). Within cycles, prediction abilities were generally high, with a trend that free threshing percent, seed mass and shattering had higher average within-site prediction compared to agronomic traits like spike yield, plant height, and spikelets per inflorescence.

**TABLE 2:** Within-site fivefold cross-validation genomic selection predictions for intermediate wheatgrass traits.

		Free Threshing%		Plant Height		Seed Mass		Shattering		Spikelets per Inflorescence		Spike Length		Spike yield	
Training Cycle	Year	*r*	sd	*r*	sd	*r*	sd	*r*	sd	*r*	sd	*r*	sd	*r*	sd
UMN-C1	2012	0.85	0.03	0.76	0.05	0.79	0.04			0.8	0.04	0.7	0.05	0.76	0.04
	2013	0.85	0.03	0.72	0.05	0.76	0.04			0.77	0.04	0.74	0.04	0.79	0.03
UMN-C2	2014	0.72	0.05	0.73	0.05			0.82	0.03			0.86	0.03	0.76	0.04
	2015	0.85	0.03	0.76	0.04			0.79	0.04			0.71	0.05	0.68	0.06
TLI-C6	2016	0.96	0	0.89	0.01	0.95	0	0.97	0	0.93	0.01	0.9	0.01	0.94	0.01
	2017	0.93	0.01	0.81	0.02	0.93	0.01	0.95	0.01	0.92	0.01	0.91	0.01	0.91	0.01
TLI-C7	2018	0.35	0.06	0.92	0.01	0.87	0.02	0.91	0.01	0.92	0.01	0.88	0.02	0.91	0.02
	2019	0.91	0.01	0.88	0.01	0.92	0.01	0.93	0.01	0.92	0.01			0.9	0.01
TLI-C8	2019	0.79	0.03	0.84	0.02	0.78	0.03	0.85	0.02	0.11	0.07			0.84	0.02

*Prediction abilities are reported as correlation (r) between predicted value and phenotypic best linear unbiased estimator (BLUP), along with the standard deviation (sd) of 100 random iterations.*

#### Across-Cycle Predictive Ability

**FIGURE 2:**
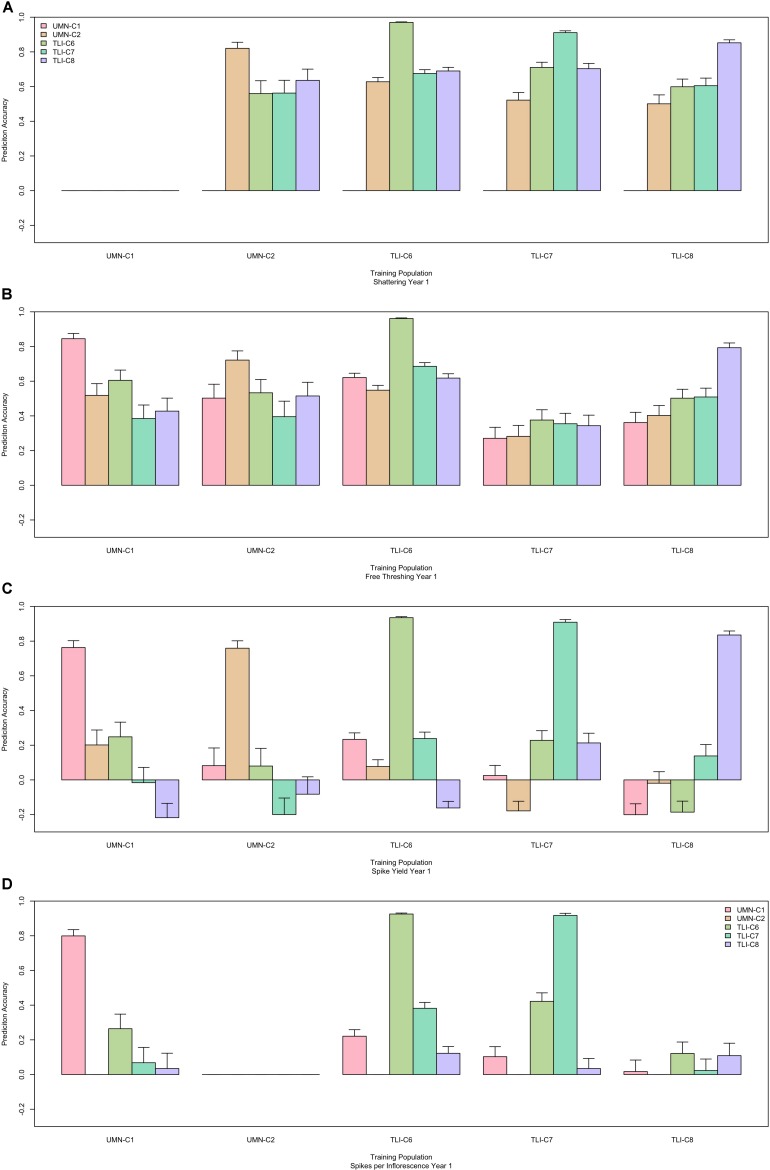
Performance of genomic selection (GS) across five cycles. Each panel represents one trait, shattering **(A)**, free threshing **(B)**, spike yield **(C)**, and spikelets per inflorescence **(D)**. The x-axis is the cycle that was used as the prediction population. Colored bars represent the prediction ability for each of the four other cycles, where each cycle forms the training population. For comparison, the fivefold cross-validation within cycle is represented for each training and prediction cycle, which usually provides the highest predictive ability. The y-axis is the prediction ability which is the correlation between the GS predicted value and the phenotypic best linear unbiased predictor (BLUP). Error bars represent the 95% confidence interval for the correlation value.

After confirming that GS could accurately predict traits within cycles, we fit GS models to predict across cycles. For each trait, all cycles were used individually as the training population, and then all other cycles were predicted from the chosen training population. This resulted in predicting each cycle from four different cycles. Across all traits, prediction ability ranged from −0.22 to 0.76, but there were striking differences between traits. For key domestication traits there was relatively high predictive ability with seed shattering in a range of 0.50–0.74, and free threshing had a range of 0.27–0.75. In comparison the agronomic trait of spike yield had a much lower range from -0.22 to 0.26 (Figure 2). These traits represent a general trend that was seen among all traits and years, allowing further discussion to be defined to domestication and agronomic traits. All other traits are provided in Supplementary Figure S1. Additionally for a trait with high and low predictive ability, scatter plots of predicted versus observed values are provided in Supplementary Figure S2.

**FIGURE 3:**
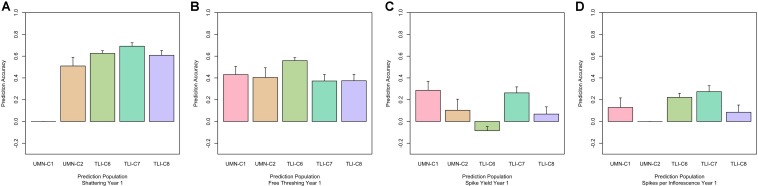
Genomic selection (GS) performance for shattering, free threshing, spike yield, and spikelets per inflorescence, **(A–D)**, respectively. Within each panel the x-axis is grouped by cycle name. Predictions were made by leaving out the named cycle and predicting that cycle from all other data. The prediction ability is the correlation between the predicted GS value and the phenotypic best linear unbiased predictor (BLUP), with standard error bars representing the 95% confidence interval.

**FIGURE 4:**
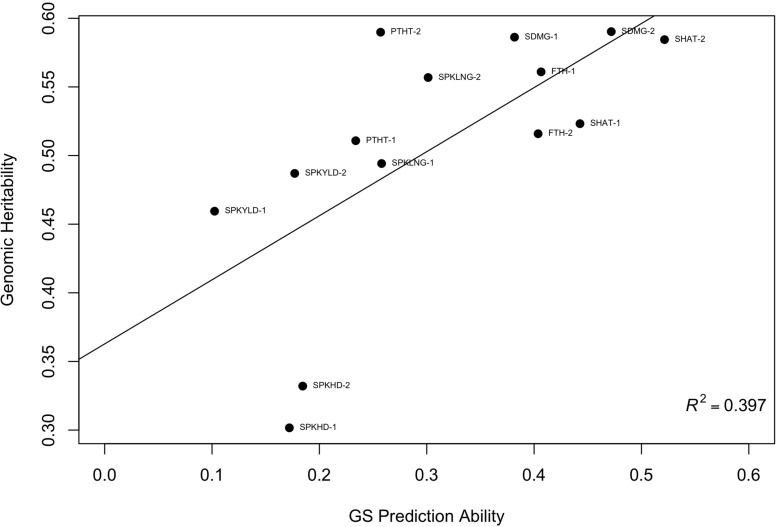
Scatter plot of genomic selection predictive ability and genomic heritability for 12 traits. For each point the trait name is provided with 1 or 2 representing year of observation where SPKYLD is spike yield; PTHT, plant height; SPKLNG, spike length; SDMG, seed mass; SPKHD, spikelets per inflorescence; SHAT, shattering; FTH, free threshing.

To further investigate the validity of the across-environment GS results, we developed GS models that used all cycle data except for the prediction set. This resulted in a larger training population which could increase GS accuracy. Prediction accuracy based on all other sites ranged from 0.35 to 0.77 for domestication traits (Figure 3). Agronomic traits such as spike yield ranged from -0.10 to 0.37 (Table 3). The predictions from this leave-one-out strategy were paired with the genomic heritability that was calculated from the GS models. Plotting these two values showed a significant relationship between these variables (*p* < 0.001, Figure 4). Key domestication traits of shattering, free threshing, and seed mass showed high heritability across cycle predictions. In comparison, spike yield, spikes per inflorescence, and plant height had lower heritability estimates and prediction accuracies.

**TABLE 3:** Genomic selection prediction abilities of intermediate wheatgrass traits across sites.

		Free Threshing%		Plant Height		Seed Mass		Shattering		Spikelets per Inflorescence		Spike Length		Spike Yield	
Prediction Site	Year	*r*	CI	*r*	CI	*r*	CI	*r*	CI	*r*	CI	*r*	CI	*r*	CI
UMN-C1	2012	0.43	0.07	0.25	0.08	0.54	0.06			0.13	0.09	0.22	0.08	0.29	0.08
	2013	0.58	0.06	0.32	0.08	0.42	0.07			0.10	0.09	0.42	0.07	0.37	0.07
UMN-C2	2014	0.40	0.08	0.42	0.08			0.51	0.07			0.20	0.10	0.10	0.10
	2015	0.42	0.08	0.48	0.08			0.61	0.06			0.46	0.08	0.12	0.10
TLI-C6	2016	0.56	0.03	0.02	0.04	0.51	0.03	0.63	0.02	0.22	0.04	0.34	0.03	–0.08	0.04
	2017	0.71	0.02	0.22	0.05	0.67	0.03	0.64	0.03	0.39	0.04	0.42	0.04	–0.10	0.05
TLI-C7	2018	0.37	0.06	0.45	0.04	0.53	0.05	0.69	0.03	0.27	0.05	0.48	0.04	0.26	0.05
	2019	0.77	0.02	0.28	0.05	0.59	0.04	0.69	0.03	0.48	0.04			0.05	0.06
TLI-C8	2019	0.37	0.06	0.29	0.06	0.35	0.06	0.61	0.04	0.08	0.07			0.07	0.07

*Prediction population was one cycle, with the training population comprising all other cycles. Predictive ability is reported as correlation between predicted value and phenotypic best linear unbiased predictor (BLUP) with ± range for the 95% confidence interval for correlation.*

#### Optimizing GS Prediction and Training Set

**FIGURE 5:**
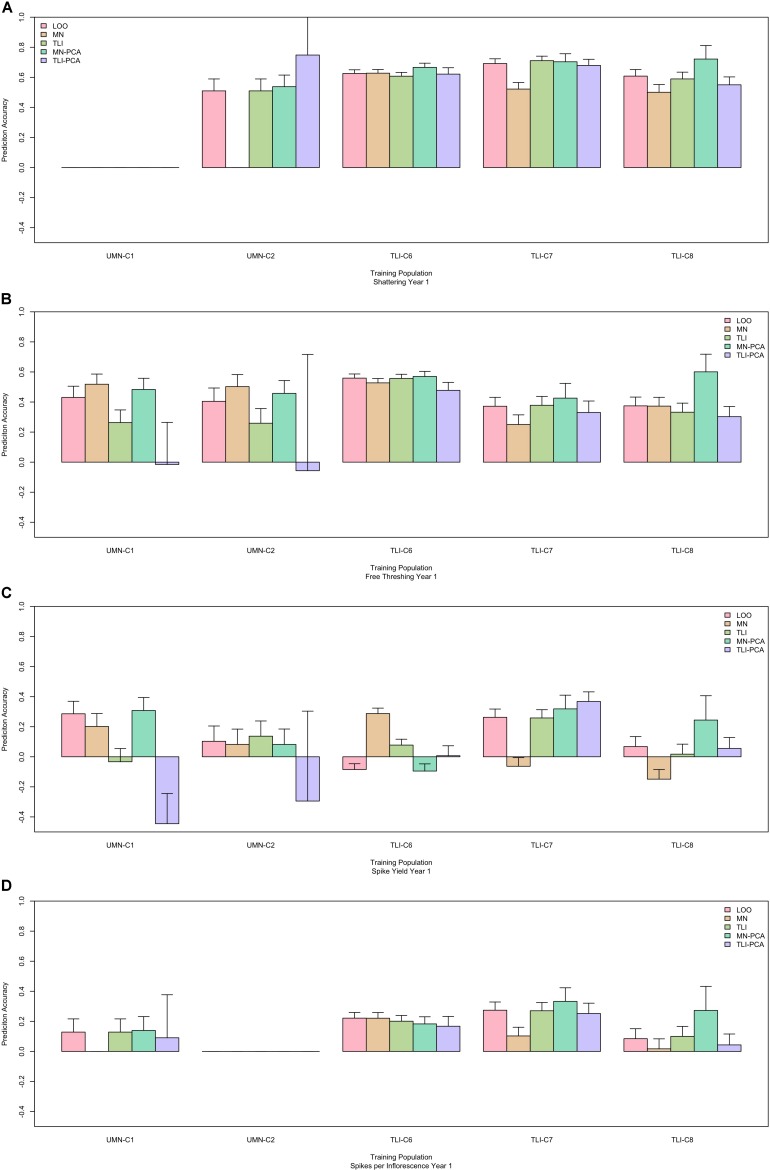
Performance of genomic selection (GS) across five cycles with different training populations. Each panel represents one trait, shattering **(A)**, free threshing **(B)**, spike yield **(C)**, and spikelets per inflorescence **(D)**. Within each panel the x-axis is grouped by cycle name. Predictions were made by: LOO, leave one out where all data other than the predicted cycle were used in the training population. MN or TLI where only data from each separate breeding program, Minnesota or Kansas respectively, were used as the training population. MN-PCA or TLI-PCA where principal component analysis (PCA) was used to cluster genets within breeding programs, MN or TLI, and form the training populations. The prediction ability is the correlation between the predicted GS value and the phenotypic best linear unbiased predictor (BLUP), with standard error bars representing the 95% confidence interval.

Finally, in an effort to determine ideal GS training populations and enhance GS results, we used two different sub-setting methods. The first subset utilized results from the PCA decomposition of the genomic marker matrix to develop two subpopulations based on relatedness, Figure 1. The second sub-setting method used each individual breeding program as a unique training population. Using these data sets, we evaluated the same across-environment GS models, with the GS training population being more closely related to the prediction population. The GS model using all cycles in a leave-one-cycle-out method, with all other cycles in the training population (Figure 3), was used as the reference. A model was declared better than the reference if the 95% confidence intervals were non-overlapping. We tested five different training populations for each of 55 cycle/trait combinations. The top performing model for each combination is listed in Table 4. Overall, there was much inconsistency between the best performing model and each cycle/trait combination (Figure 5 and Supplementary Figure S3). However, using the leave-one-out as a reference resulted in the best performing model 62% of the time (34 of 55 combinations).

**TABLE 4:** Highest performing genomic selection (GS) model for each trait/cycle combination across five breeding cycles representing two different breeding programs.

Prediction site	Year	Free threshing	Plant height	Seed mass	Shattering	Spikelets per inflorescence	Spike length	Spike yield
UMN-C1	2012	MN^†^	LOO	TLI-PCA		LOO	KS	LOO
	2013	LOO	LOO	LOO		TLI-PCA	LOO	LOO
UMN-C2	2014	MN	MN		LOO		KS	LOO
	2015	LOO	LOO		LOO		LOO	MN
TLI-C6	2016	LOO	KS	LOO	UMN-PCA	LOO	LOO	MN
	2017	LOO	LOO	LOO	LOO	LOO	LOO	MN
TLI-C7	2018	LOO	LOO	TLI-PCA	LOO	LOO	LOO	TLI-PCA
	2019	LOO	LOO	KS	TLI-PCA	LOO		KS
TLI-C8	2019	UMN-PCA	LOO	LOO	UMN-PCA	UMN-PCA		UMN-PCA

*Predictive ability was assessed as the correlation between the GS predicted value and the phenotypic best linear unbiased predictor (BLUP). Models differed with respect to the training population used to develop the model. The leave one-out, LOO, model was used as the reference model and only if a model exceeded the 95% confidence interval of the LOO model was it considered superior. ^†^Models are: LOO, leave-one-out, prediction cycle is left out of the training set, and all other cycles are used to train the model. MN and KS are breeding-program specific where only genets from Minnesota (or Kansas) are used to predict each cycle. For TLI-C6 2016 plant height, KS training population would consist of TLI-C7 and TLI-C8, with TLI-C6 as the prediction population. UMN-PCA and TLI-PCA are where the training population is made from PCA analysis of the marker matrix, with UMN-PCA encompassing most UMN lines and some of TLI that were more similar to UMN material than the TLI subset.*

## Discussion

### Combining Data Resources

The affordability of next-generation sequencing provides many opportunities for breeding that were previously unavailable. Particularly for programs that are implementing GS, there is an opportunity to leverage data across breeding programs and identify synergistic opportunities. This is particularly the case for minor and emerging crops. We were able to combine five cycles representing nearly a decade of breeding progress for IWG in the Central USA. Across the two programs, many key traits were measured each year, but there were often minor differences in trait measurement, specifically scoring of free-threshing and total spike yield between the TLI and UMN programs. While our results did not show any marked difference in these traits, i.e., consistent free-threshing prediction and inconsistent spike yield across other cycles, it is unknown if more consistent data collection would result in higher predictive ability within this data set. As other breeding programs are established trait standardization using crop ontology (Shrestha et al., 2010) could greatly increase the inter-operability of experimental data.

### Genomic Selection Accuracy and Analysis

#### Within-Cycle Predictive Ability

Using data generated from the field trials and next generation sequencing, we evaluated the potential of GS to predict trait values across geographically distant IWG breeding programs. First, within-cycle predictions were generated to verify GS could appropriately predict trait values (Table 2). These cross-validation predictions were the highest GS predictive abilities achieved because the training sets were highly related and the training and test sets were grown in the same environment, minimizing any genotype by environment interactions (Desta and Ortiz, 2014; Zhang et al., 2016). These prediction abilities provide a potential maximum value that could be achieved utilizing the current markers and phenotypes within the study. Additionally, these predictions show that within breeding programs, GS could be an effective way to enhance genetic gain in IWG.

#### Across-Cycle Predictive Ability

After evaluating within-site GS prediction, across-site predictions were generated for all cycles. As the relatedness and environments changed, a decrease in GS predictive ability was observed. Within these evaluations, two general trends emerged. For key domestication traits such as shattering and free-threshing, GS predictions were relatively high and constant across environment (Figure 2). For agronomic and yield related traits, the results were inconsistent, with some sites even producing negative prediction abilities. This suggests that certain traits may be more amenable to multi-environment GS than other traits.

To further investigate this trend, we examined the genomic heritability from the GS models. Plotting genomic heritability and predictive ability (Figure 4) suggests that domestication traits may exhibit lower genotype-by-environment interaction than agronomic traits. Additionally, the resulting prediction abilities of various traits were reaching the level of the trait heritability.

Domestication traits were highly predictive across environments, possibly indicating that these traits are not as influenced by environment as other traits. Within wheat, there are several well-known genes that control these traits. Free threshing in wheat is determined by a recessive mutation in the *Tg* (*tenacious glume*) locus and the dominant mutation of the *Q* gene. The *Tg* loci has been reported to explain up to 44% of the variation in threshability, and at least five other quantitative trait loci (QTLs) for threshability have been observed in wheat (Jantasuriyarat et al., 2004). Within IWG, recent research by Larson et al. (2019) found that QTL markers explained up to 46% of variation for free threshing across two locations. The *Br* (*brittle rachis*) locus controls shattering in wheat with two dominant genes and is homoeologous to the *Btr* loci in barley (Nalam et al., 2006). Traits such as free threshing and shattering that may have larger-effect QTL could be both increasing GS predictions as well as maintaining predictive ability across environments.

For agronomic traits, many more QTL of much smaller size have been reported. Bajgain et al. (2019) identified over 154 QTL for seven agronomic traits in IWG with the largest QTL effect sizes explaining only 4% of the phenotypic variation. Larson et al. (2019) found 12 QTL that explained up to 27% of the variation of spike yield in a biparental population grown in five environments. As the number of QTL increase and their size decreases, adequately accounting for their effects across environments may be more challenging. Simulation studies have shown that as heritability decreases GS accuracies are lowered (Iwata and Jannink, 2011). Other research has indicated that GS accuracy diminishes as the number of QTL increases (Shengqiang et al., 2009).

#### Optimizing GS Prediction

Complementary to evaluating how traits may respond to GS, we also examined how the training population could be optimized to achieve the best results when combining data across breeding programs. While all germplasm originated from TLI material, UMN-C1 was only a subset of the entire TLI program and UMN-C2 was selected for MN conditions, which are different than KS. Additionally, from the founding lines (TLI-C3) two and five generations of selection had occurred for UMN and TLI respectively, allowing for potential population divergence.

We evaluated models using a leave-one-out approach for all cycles, which should result in the largest training population available for GS prediction. This leave-one-out strategy insured that the models were not biased by the size or the relationship of the training population (Desta and Ortiz, 2014) in comparison to GS prediction made from individual cycles. Additionally, we used PCA to develop a subset of data more related to each breeding program to ensure any large population structure differences did not influence GS prediction (Norman et al., 2018). Finally, data predictions were also developed using data specific to each breeding program.

The results from these models were inconsistent, with the leave-one-out model performing as well as or better the majority of the time. Often breeding program-specific or PCA-specific subsets performed well, but there was no clear pattern to this performance (Table 4). For example, the optimized training set using PCA for UMN provided the best prediction for TLI-C8 free threshing, whereas the TLI-PCA optimized training set provided the best prediction for UMN-C1 seed mass. In this case the training sets had optimal performance in data sets for which they were not specifically optimized. While developing highly optimized prediction sets has been shown to increase prediction accuracies (Isidro et al., 2015; Rutkoski et al., 2015) we did not note this in this data set. This could result from the large amount of genetic variance compared to domesticated crops. While future breeding efforts may be enhanced by optimizing the training set, these data suggest that increasing the training population, i.e., leave-one-out, is generally more useful than optimizing relatedness to prediction candidates.

### Implication for Future Development

As concerted efforts to develop new crops through domestication of crop wild relatives continues for food security and environmental benefits (Glover et al., 2010; Mayes et al., 2012), we have evaluated approaches for genomics-assisted breeding of neo-domesticated crops with insights into maximizing genetic gains. While plant breeding is both expensive and time consuming (Crews and DeHaan, 2015; DeHaan et al., 2016), genomic technologies provide a way to accelerate compared to phenotypic selection (Varshney et al., 2012; Unamba et al., 2015). Next-generation sequencing coupled with powerful tools such as GS and genome wide association studies could allow for significantly improving agronomic and domestication traits in short periods of time, especially in non-model plants. Within the TLI-IWG breeding program, GS has reduced the breeding cycle time from 2 years to 1 year, which should effectively double the rate of genetic gains if the predictability is roughly equivalent to the narrow-sense heritability. Additionally, the genetic resources generated can be used to better understand the genetic architecture of important agronomic and domestication traits (examples include Bajgain et al., 2019; Larson et al., 2019).

These results show that as plant species undergo early domestication, collaboration will accelerate progress, i.e., not every breeding program will have to solve the same domestication problems and that progress can be made across programs. As domestication traits are fixed, breeding programs can work toward developing adapted lines for targeted growing regions. DeHaan et al. (2016) suggest a pipeline strategy for new crop domestication where many candidates are tested and attrition occurs as information about candidates are gained. Cooperative efforts in early breeding stages, along with applied genomics, should result in more quickly advancing and developing promising species into commercially viable crops.

These data provide several potential use cases for breeding programs. If a program is beginning, there appears to be little downside in utilizing training data sets from across programs. As programs mature and have sufficient, data from multiple years and locations, GS models can be developed within programs. This could be especially important for agronomic traits such as spike yield as combining data across programs could result in negative predictions (Figure 5). However, when looking at GS models using program-specific data, the GS predictions were always positive, so program-specific models may be the most conservative way to insure genetic gains. For domestication traits, predictions were usually similar regardless of the training population, suggesting minimal benefit to pooling multiple locations.

Our results show that GS can be a powerful tool in breeding programs, yet GS is not a single, stand-alone solution for quickly developing new crops. While we envision GS improving with larger data sets and new statistical model development, multi-environment predictions are extremely complex. To fully leverage genomic resources, GS should be integrated with phenomic and environmental data. High-throughput phenotyping is an emerging field that is providing dense phenomic measurements (White et al., 2012; Araus et al., 2018) that have been shown to increase GS model accuracy (Rutkoski et al., 2016; Crain et al., 2018). A further complement to better predict how the environment influences phenotype will include incorporating crop models to better understand plant development within a range of environments (i.e., review of crop models in wheat by Chenu et al., 2017). Future advances in these areas as well as incorporating them into unified prediction models will allow scientist to drive genetic gain in novel crops across a range of environments.

## Conclusion

Domesticating crop wild relatives is a challenging and time consuming task (Cox et al., 2002; DeHaan et al., 2014). Previous research at TLI has shown that a 77% increase in seed yield was achieved in two cycles of selection, however, to reach yields of annual wheat another 20 years of sustained breeding gains would be required with even longer time intervals to achieve similar seed mass to wheat (DeHaan et al., 2014).

Perennial grains derived from the domestication of wild species hold much promise for environmental and human benefit. To achieve these benefits, specific traits of wild species will need to be modified. Within IWG, free-threshing and non-shattering seed types are two key domestication traits that must be improved for wide-scale adoption. In addition, the economic yield of IWG must be sufficient to incentivize the transition to new crops. Along with fixing key traits for domestication, breeding efforts should also ensure that crops are broadly adapted (DeHaan et al., 2016).

The ability to use molecular tools such as GS, combined with modern breeding methodologies, may allow perennial crops and crop wild relatives to compress the 10,000 year selection history of many annual crops into a few decades. While GS predictions for agronomic traits like spike yield were low between breeding sites and environments, significant synergies could be achieved by utilizing collective information about domestication traits. While site-specific or regional programs will be necessary to breed for the best locally adapted genets, progress made toward improving key domestication traits could be shared among all programs. This is especially important for resource-limited programs that are domesticating new crops, allowing improvement for traits that are less environmentally influenced and are essential for domestication. Early domestication work could be carried out by a single program or shared among programs with each program phenotyping a few lines in diverse locations to quickly and efficiently improve key traits. As more programs are initialized for the breeding of IWG, they will be able to identify germplasm that has key domestication traits and be able to focus breeding efforts toward achieving higher site-specific agronomic performance.

## Data Availability Statement

The genotypic datasets analyzed for this study have been placed in the NCBI Sequence Read Archive (SRA) (https://www.ncbi.nlm.nih.gov/bioproject/) BioProject accession numbers PRJNA563706, PRJNA609095, and PRJNA608473. All phenotypic data and scripts for data analysis have been placed in the Dryad Digital Repository (https://doi.org/10.5061/dryad.3j9kd51d9).

## Author Contributions

JC, LD, and PB conceived experimental ideas and methods. LD, JA, XZ, and PB conducted all field evaluations. LD, JP, PB, and XZ performed DNA extraction and genotyping. JC completed data analysis and wrote the manuscript. All authors read, reviewed, and approved the final manuscript.

## Conflict of Interest

The authors declare that the research was conducted in the absence of any commercial or financial relationships that could be construed as a potential conflict of interest.

## Footnotes

1 https://phytozome-next.jgi.doe.gov/info/Tintermedium_v2_1

## Supplementary Material

FIGURE S1 Performance of genomic selection (GS) across five cycles. Each panel represents one trait, A-N. The x-axis is the cycle that was used as the prediction population. Colored bars represent the prediction ability for training populations for other cycles. For comparison, the within cycle fivefold cross-validation is represented for each training and prediction site, which usually provides the highest predictive ability. The y-axis is the prediction ability which is the correlation between the GS predicted value and the phenotypic best linear unbiased predictor (BLUP). Error bars represent the 95% confidence interval for the correlation value.

FIGURE S2 Relationship of genomic selection (GS) predicted values (x-axis) and observed values (y-axis) for two traits, including line of best fit. In panel A, a free threshing (n = 2,496), a trait with high predictive ability is shown, while panel B represents a trait with low predictive ability, spike yield (n = 2,508). The training population was TLI-C7 and the prediction population was TLI-C6.

FIGURE S3 Genomic selection (GS) performance across five cycles where each panel represents one trait A-N. Within each panel the x-axis is grouped by the cycle of data that was predicted, with different training populations represented by colored bars. Leave-one-out (LOO) was a training population where the cycle of interest was left out and all other cycles were used to predict the cycle. Breeding program-specific training populations were developed for the Minnesota (MN) and Kansas (KS) breeding programs. Finally, UMN-PCA and TLI-PCA are training populations that were developed using principal component analysis (PCA) of the marker matrix. UMN-PCA is a training population more closely related to UMN genets while TLI-PCA is more closely related to TLI genets. The prediction ability is the correlation between the predicted GS value and the phenotypic best linear unbiased predictor (BLUP), with standard error bars representing the 95% confidence interval.

## Abbreviations

BLUPs: best linear unbiased predictors
GBS: genotyping-by-sequencing
GS: genomic selection
IWG: intermediate wheatgrass
PCA: principal component analysis
QTL: quantitative trait loci
SNP: single nucleotide polymorphism
TLI: The Land Institute.
