# Supplementary figures and images for: Enhancing Crop Domestication Through Genomic Selection, a Case Study of Intermediate Wheatgrass
Source: Front Plant Sci. 2020 Mar 24;11:319. doi: 10.3389/fpls.2020.00319 (PMC7105684; doi:10.3389/fpls.2020.00319)

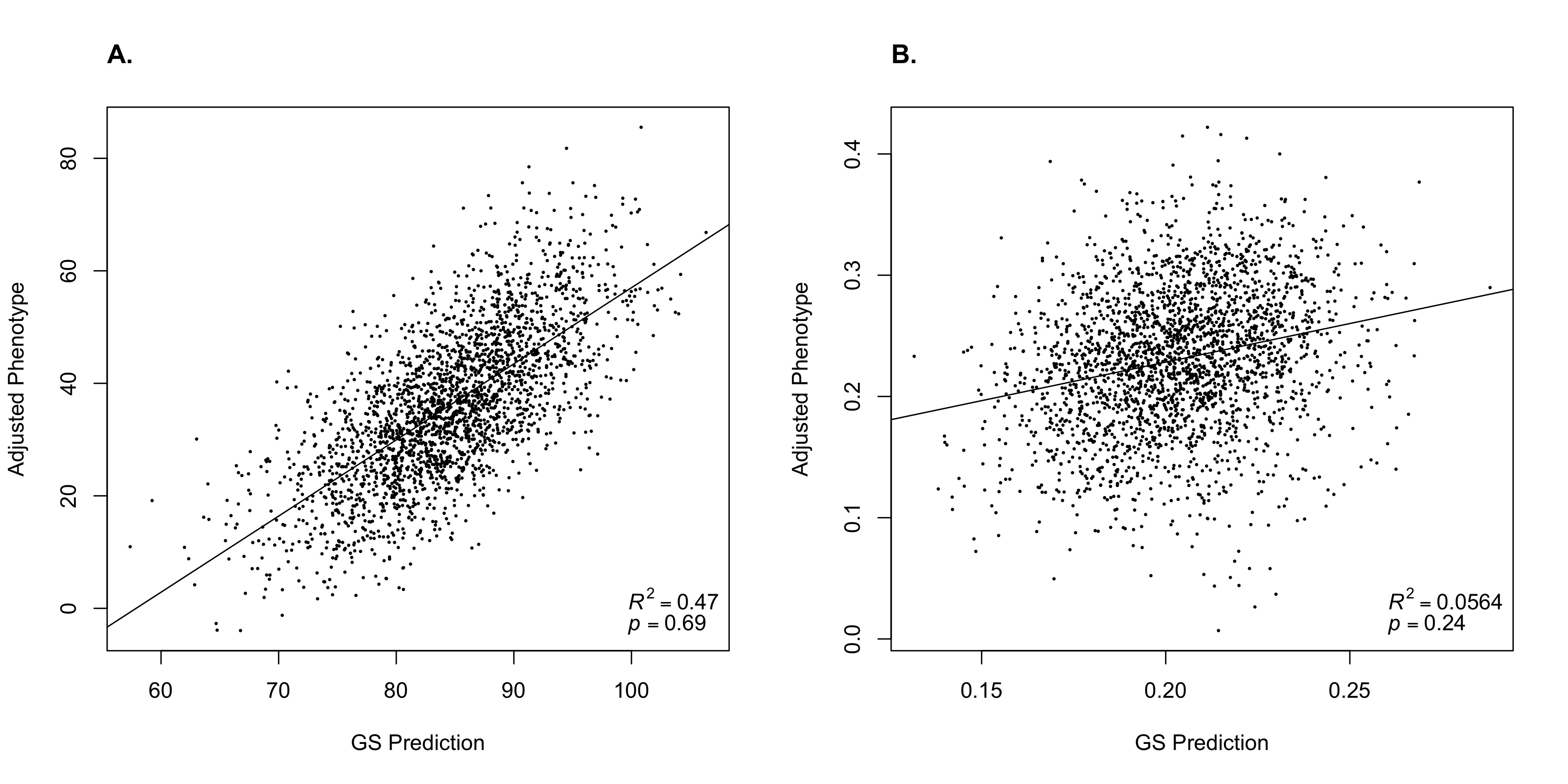

Supplement: FIGURE S2 — Relationship of genomic selection (GS) predicted values (x-axis) and observed values (y-axis) for two traits, including line of best fit. In panel A, a free threshing (n = 2,496), a trait with high predictive ability is shown, while panel B represents a trait with low predictive ability, spike yield (n = 2,508). The training population was TLI-C7 and the prediction population was TLI-C6. [file Image_2.JPEG]
